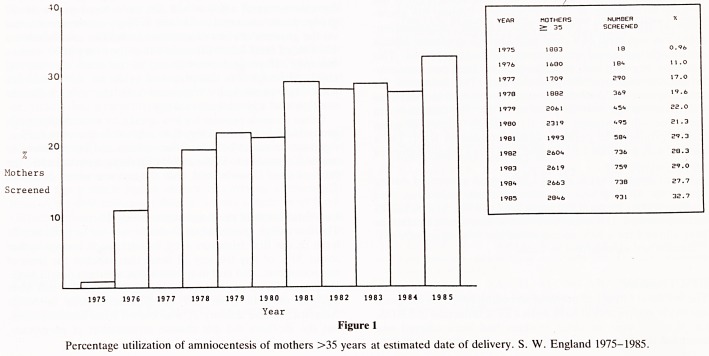# The Prevention of Down's Syndrome in the South Western Region of England 1975–1985

**Published:** 1990-03

**Authors:** Nigel Wilson, Daisy Bickley, Alan McDermott

**Affiliations:** Department of Child Health, University of Bristol; South Western Regional Cytogenetics Centre, Southmead Hospital, Bristol; South Western Regional Cytogenetics Centre, Southmead Hospital, Bristol

**Keywords:** DOWN'S SYNDROME, PRENATAL DIAGNOSIS, AMNIOTIC FLUID, CHROMOSOMAL ABNORMALITIES

## Abstract

Cytogenetic prenatal screening for Down's syndrome in the South West Region of England from 1975 to 1985 was reviewed. The use of amniocentesis increased, and for the years 1981 to 1985 averaged 29.4% of women 35 years or over at their estimated date of delivery. 58 pregnancies were terminated after karyotyping of amniotic fluid cells confirmed trisomy 21.

385,440 live births were born in the region, 452 with Downs's syndrome, giving a live birth incidence of 1 in 853. The effective impact of prenatal screening was calculated at an overall 8.3% reduction in Down's syndrome live births, but for the years 1981 to 1985 this rose to 11.3%.

In spite of the introduction of new prenatal screening programmes that are not reliant solely on maternal age, it is predicted that substantial numbers of children with Down's syndrome are likely to be born each year. Adequate medical facilities will still be required for the survivors.


### The Prevention of Down's Syndrome in the South Western Region of England 1975–1985

**Nigel Wilson, MRCP**
Department of Child Health, University of Bristol

**Daisy Bickley MA, Alan McDermott PhD, MRCPath**
South Western Regional Cytogenetics Centre,
Southmead Hospital, Bristol
Correspondence to: Dr A McDermott Director, S.W. Regional Cytogenetics Centre, Southmead Hospital, Bristol BS10 5NB

### ABSTRACT
Cytogenetic prenatal screening for Down's syndrome in the South West Region of England from 1975 to 1985 was reviewed. The use of amniocentesis increased, and for the years 1981 to 1985 averaged 29.4% of women 35 years or over at their estimated date of delivery. 58 pregnancies were terminated after karyotyping of amniotic fluid cells confirmed trisomy 21.

385,440 live births were born in the region, 452 with Downs's syndrome, giving a live birth incidence of 1 in 853. The effective impact of prenatal screening was calculated at an overall 8.3% reduction in Down's syndrome live births, but for the years 1981 to 1985 this rose to 11.3%.

In spite of the introduction of new prenatal screening programmes that are not reliant solely on maternal age, it is predicted that substantial numbers of children with Down's syndrome are likely to be born each year. Adequate medical facilities will still be required for the survivors.

### KEY WORDS
DOWN'S SYNDROME
PRENATAL DIAGNOSIS
AMNIOTIC FLUID
CHROMOSOMAL ABNORMALITIES

### INTRODUCTION
Cytogenetic prenatal detection of Down's syndrome using mid-trimester amniocentesis was introduced in the South Western (S.W.) Region in the mid-1970s. The programme was based on maternal age because of the observation that increased maternal age was associated with increased risk of chromosomal abnormality (1).

A study was undertaken to review the impact of this programme in the South West, and to relate these findings to national and international programmes.

### Methods
All chromosomal analyses from South West England are undertaken at the one central laboratory: the Regional Cytogenetics Centre at Southmead Hospital, Bristol. All prenatal cytogenetic studies are registered and records are available on maternal age, expected date of delivery (EDD), hospital of referral, but not maternal place of residence.

From 1975 the Regional Laboratory agreed, when requested, to undertake cell culture and karyotyping on amniotic fluid from women aged 35 or more at the time of their EDD. When trisomy 21 was detected prenatally, the outcome of the pregnancy was recorded by the cytogenetics centre; these records were reviewed for the years 1975–1985. Records of all births and stillbirths by maternal age and residency for the years studied were obtained from the Office of Population Censuses and Surveys (OPCS) (2). Utilization rates of prenantal screening by women over 35 years could thus be calculated, and expressed as a percentage.

There is no central registry of either those individuals with mental handicap, or those with Down's syndrome in the S.W. Region. All children born suspected of having Down's syndrome and having chromosomal analysis, were karyotyped at the Regional Centre. Age of infant at chromosomal analysis, maternal age, name of hospital and the referring paediatrician were recorded. It was thus possible to estimate the incidence of live born Down's syndrome (LBDS).

For the years 1981–1985, a cross-check of LBDS children was made by obtaining information from the eleven health districts in the Region using questionnaires sent to the senior clinical medical officers concerned with mental handicap.

| YEAR | MOTHERS ≥ 35 | NUMBER SCREENED | % |
|---|---|---|---|
| 1975 | 1883 | 18 | 0.96 |
| 1976 | 1680 | 184 | 11.0 |
| 1977 | 1709 | 290 | 17.0 |
| 1978 | 1882 | 369 | 19.6 |
| 1979 | 2061 | 454 | 22.0 |
| 1980 | 2319 | 495 | 21.3 |
| 1981 | 1993 | 584 | 29.3 |
| 1982 | 2604 | 736 | 28.3 |
| 1983 | 2619 | 759 | 29.0 |
| 1984 | 2663 | 738 | 27.7 |
| 1985 | 2846 | 931 | 32.7 |

**Figure 1**

Percentage utilization of amniocentesis of mothers >35 years at estimated date of delivery. S. W. England 1975–1985.

### RESULTS

From 1975–1985, 452 children born in the S.W. Region were confirmed as genotype trisomy 21. This included 37 children with mosaic or translocation forms with phenotypes which were sufficiently suggestive of Down's syndrome for chromosome analysis to have been requested. Of the 452 LBDS children, 436 were karyotyped in the neonatal period and only 16 (3.3%) at a later date.

There were 385,440 live births in the area in the time period giving an incidence of chromosomally proven LBDS of 1/853. This is the incidence after any impact from the prenatal screening programme.

During the period studied, the proportion of live births to older mothers was between 5.1% to 7.5% of all live births. The utilization rate of amniocentesis by pregnant women of 35 years or more, increased over the study period (Fig 1). In the latter 5 years 3,748 amniocenteses were performed from 12,725 women 35 years or older achieving a 29.4% utilization rate. This represents 2.1% of all pregnancies. The service was used by 24.3% of the 35–39 years old group and 46.5% of the 40 year plus group.

**Table 1**

**Number of live birth Down's Syndrome and number of terminations of pregnancy of trisomy 21. S. W. England 1975–1985.**

| *Year* | *LBDS* | *D. S. Terminations* |
|---|---|---|
| 1975 | 36 | 1 |
| 1976 | 29 | 2 |
| 1977 | 44 | 3 |
| 1978 | 48 | 5 |
| 1979 | 50 | 4 |
| 1980 | 32 | 5 |
| 1981 | 39 | 5 |
| 1982 | 54 | 7 |
| 1983 | 40 | 12 |
| 1984 | 45 | 6 |
| 1985 | 35 | 8 |
| | 452 | 58 (41 prevented*) |

$$\frac{41}{493} = 8.3\% \text{ prevented}$$

* Allows for 30% late spontaneous fetal death rate.

From 1975–1985, 58 pregnancies were electively terminated after trisomy 21 had been confirmed after amniocentesis for all age groups (Table 1). Two other women chose not to have termination of their pregnancy, despite the knowledge of trisomy 21. Due to maternal cell contamination, there was one birth of a child with Down's syndrome after a normal chromosomal complement had been reported. This one false negative test, together with no false positives confirmed the high specificity of the test. The indication for amniocentesis in 55 of the 58 women who had terminations was maternal age over 35 years at EDD. The indications in the three other women were (i) a family history of spina bifida, primarily to perform alpha fetoprotein level, (ii) a mother with previous LBDS child, and (iii) a family history of mental retardation.

It is estimated that 8.3% of LBDS were prevented during the years 1975–1985. As amniocentesis utilization had reached higher levels in the years 1981 to 1985, 11.3% LBDS were prevented. It is important to note that these calculations have allowed for a 30% spontaneous late fetal death rate (3).

Results are summarised in Table 2.

**Table 2**

**Summary of results of cytogenetic prenatal screening for Down's Syndrome, S. W. Region of England 1975–1985.**

| | |
|---|---|
| *1975–1985* | |
| Live Births | 385,440 |
| Live Births Down's syndrome (LBDS) | 452 |
| Incidence LBDS | 1/853 |
| % Livebirths to mothers >35 years | 5.1–7.5% |
| Terminations of pregnancy for Trisomy 21 | 58 |
| Prevented LBDS * | 8.3% |
| *1981–1985* | |
| No. Livebirths to women >35 years | 12,725 |
| No. amniocenteses | 3,748 |
| Utilization rate | 29.4% |
| % amniocentesis of Live births | 2.1% |
| Prevented LBDS* | 11.3% |

### DISCUSSION

The maximal impact of prenatal screening based on maternal age in the region would have been a 29% reduction in LBDS, i.e. if all 35 year or older mothers had been offered and accepted the test and its implications. This theoretical 29% reduction of LBDS is similar to 29% in Scotland (4), 30% in New York state (5), and 27% in Western Australia (6). Conversely 71% of LBDS would never have been prevented by this programme in the S.W. Region based on maternal age.

The 29.4% utilization rate of women greater than 35 years old in the era 1981–1985 was similar to New York (5), Western Australia (6) and Mersey and Wales (7). It is pertinent to record that data for the three years subsequent to the study show an increase in utilization of the 35–39 year old women to 28%, and in the 40 year and older women to 56% (33% overall).

Comparisons of a one year survey in 1984 of all regions in the United Kingdom showed in the 35–39 year group, uptake varied between 16% to over 40%, and in the 40 year plus group, uptake was between 27%–63% (8). The S.W. Region figures are in the average range for the country, and hence may better reflect national trends in amniocentesis usage than those reported from Mersey (7). The S.W. Region figures are based on regional referrals and not on maternal residency. The Association of Clinical Cytogeneticists recognizes the substantial cross-flow between regions, but South West England was not mentioned as a region with notable cross-flow (8).

One reason why utilization rates were around 25% in the 35–39 year old group is variation in age at which individual obstetricians will offer amniocentesis. Although any sample for chromosomal analysis will not be refused in the 35–37 group, many obstetricians do not actively encourage the test on the grounds of risks of amniocentesis. The usually quoted 1% risk of fetal death (9) means that for every 1000 women screened, 10 pregnancies will end in the death of a normal child. Although it is inappropriate to do so, these risks are sometimes numerically compared with the risk of Downs syndrome at a given maternal age.

Other possible reasons for low uptake by women of any age group include late booking, late referral to the obstetrician, religious or moral beliefs of the woman and her relatives or medical attendants. Some elderly mothers reported after the birth of their Down's child, that the test was never discussed (10).

### Late fetal death of the fetus with trisomy 21

There is a high spontaneous abortion rate for any trisomy 21 fetus in the first trimester (11), but less well known is that about 30% of any trisomy 21 fetus that reaches the time of amniocentesis, also end in spontaneous abortion or still birth. Dr Ernest Hook provided convincing evidence for this from analysis of data from 134 cytogenetic centres in North America. Where trisomy 21 was known from amniocentesis, but the mothers did not choose termination of pregnancy, there was a 23.8% late fetal death (3).

The corollary of late fetal death of the trisomy 21 pregnancy, is that an estimate of 30% of trisomy 21 fetuses diagnosed at amniocentesis would end in spontaneous abortion or stillbirth, and to express "prevented" births in terms of the numbers of terminations may be seen as an overestimate.

### NEW DEVELOPMENTS AND FUTURE PREDICTIONS

The association of low maternal serum alpha fetoprotein (MSAFP) as a risk factor for Down's syndrome (12) has been incorporated in screening programmes. For this review, MSAFP had no impact as it was not generally or consistently used between 1975–1985. At the time of writing, five of the 11 health districts in South West England had incorporated MSAFP into their screening programmes, and one was about to do so.

It should be noted that risk tables for Down's syndrome that have been published combining maternal age and MSAFP are cumulative, not individual risks. This means that although useful in predicting the consequences of differing screening protocols, a given point on the table will not predict accurately the individual risk for that pregnancy. It appears that there is already a reluctance by many clinicians and mothers not to undergo amniocentesis for women over 35 years, when their MSAFP suggests lower risk than for age alone (13). Many more amniocenteses could thus be needed with increased cost to the programme.

There are significant differences in the ranges of MSAFP for given risk prediction of Down's syndrome from various laboratories in the United States and Great Britain (14) and there is a lack of uniformity of the technical assays involved in measuring MSAFP levels (15). On the plus side, it has been found that when gestation is corrected by ultrasound, a number of those low MSAFP results fall in the normal range, further reducing the proportion of women for whom amniocentesis is indicated.

Other second trimester methods for the detection of Down's syndrome include low maternal serum unconjugated oestriol (MSUO) (16), which is independent of maternal age and thus complementary to age and MSAFP, and high maternal serum concentration of human chorionic gonadotrophin (MSHCG) (17). Wald et al (18) have suggested that a composite risk for a mother can be calculated from maternal age, MSAFP, MSUO, and MSHCG. They calculate that 60% of Down's syndrome pregnancies could be detected with a 5% amniocentesis rate. As 2.1% of the S.W. Region underwent amniocentesis during 1975 to 1985, a large increase would be needed in the numbers of amniocentesis performed.

Use of ultrasound in the detection of Down's syndrome pregnancies includes the measurement of nuchal skin fold (thicker than normal), femur length related to biparietal diameter (shorter than normal) (19), and the recognition of an intracardiac atrioventricular septal defect by fetal echocardiography (20).

First trimester detection of Down's syndrome by chorionic villous biopsy (21) and by maternal biochemical screening is also being investigated (22). Up to 80% of trisomy 21 fetuses abort spontaneously in the first trimester (11). The elderly mother has a much higher chance of carrying a trisomy 21 fetus in the first trimester, than at amniocentesis or at birth. Objective counselling will require that the obstetrician inform the mother clearly on both the chances of abnormality at sampling and at the time of birth.

### CONCLUSIONS

Prenatal screening based on increased maternal age has a theoretical reduction of live born Down's syndrome of 30%, but achieved 11.3% in the S.W. Region in 1981–1985, as amniocentesis became more widely used. Predictions of a 60% reduction in LBDS have been made for programmes based on biochemical parameters and maternal age, but there are reasons why a somewhat smaller reduction is more likely. Large numbers of children with Down's syndrome will continue to be born into the Region. With the life expectancy of such individuals now over 50 years in the western world (23), there will be a continued need for provision of community and specialist medical services for these individuals.

### REFERENCES

1. PENROSE, L. S. (1949) The incidence of mongolism in the general population. *J. Ment. Sci.* **95**, 685–688.
2. OPCS Publications. 1975–1985.
3. HOOK, E. B. (1978) Spontaneous deaths of fetuses with chromosomal abnormalities diagnosed prenatally. *N. Engl. J. Med.* **299**, 1036–1038.
4. HAGARD, S. and CARTER, F. A. (1976) Preventing the birth of infants with Down's Syndrome. A cost benefit analysis. *Br. Med. J.* **1**, 753–756.
5. HOOK, E. B., SCHRENEMACHERS, D. M. and CROSS, P. K. (1981) Use of prenatal cytogenetic diagnosis in New York State. *N. Engl. J. Med.* **305**, 1410–1413.
6. MULCAHY, M. T. and MICHAEL, C. A. (1983) The utilization of prenatal cytogenetic diagnosis in Western Australia. *Aust. NZ. J. Obst. Gynaec.* **23**, 8–10.
7. WALKER, S. and HOWARD, P. J. (1986) Cytogenetic prenatal diagnosis and its relative effectiveness in the Mersey region and North Wales. *Prenatal Diagnosis*. **6**(1), 13–23.
8. A. C. C. Review of clinical cytogenetic services 1984. Published by the association of clinical cytogeneticists, U.K. 1986.
9. TABOR, A., PHILLIP, J. and MASDEN, M. *et al.* (1986) Randomized controlled trial of genetic amniocentesis in 4606 low-risk women. *Lancet* **i** 1287–1293.
10. FERGUSON-SMITH, M.A. (1983) Prenatal Chromosome Analysis and its impact on the Birth Incidence of Chromosome Disorders. *Brit. Med. Bull.* (39)**4**, 355–364.
11. BOUE, J., BOUE, A. and LAZZAR, P. (1975) Retrospective epidemiological studies of 1560 karotyped spontaneous human abortions. *Tetralogy* **12**, 11–26.
12. CUCKLES, H. S., WALD, J. and LINDENBAUM, R. H. (1984) Maternal serum alphafetoprotein measurement: a screening test for Down's syndrome. *Lancet* **i**, 926–929.
13. HARRIS, R. and ANDREWS, T. (1988) Prenatal screening for Down's syndrome. *Arch Dis Child.* **53**, 705–706.
14. HOOK, E. B. (1988) Variability in predicted rates of Down's syndrome associated with maternal serum alpha-fetoprotein levels in older women. *Am J Hum Genet* **43**, 160–164.
15. KNIGHT, G. J., PALOMAKI, G. E. and HADDOW, J. E. (1988) Use of maternal alphafetoprotein measurements to screen for Down's syndrome. *Clin Obstet Gynaecol* **31**, 306–327.
16. CANNICK, J. A., KNIGHT, G. J., PALOMAKI, G. E. *et al.* (1988) Low unconjugated oestiol in pregnancies with Down's syndrome. *Brit J Obstet Gynaecol* **95**, 330–333.
17. BOGART, M. H., PANDIAN, M. R. and JONES, O. W. (1987) Abnormal maternal serum chorionic gonadotrophin levels in pregnancies with fetal chromosomal abnormalities. *Prenatal Diagnosis* **7**, 623–630.
18. WALD, N. J., CUCKLE, H. S., DENSEM, J. W. *et al.* (1988) Maternal serum screening for Down's syndrome in early pregnancy. *Brit Med J* **297**, 883–887.
19. BANACEREF, B. R., GELMAN, R. and FRIGOLETTO, F. D. (1987) Sonographic identification of second trimester fetuses with Down's syndrome. *N Engl J Med* **317**, 1371–1376.
20. SANDOR, G. G. S., FARQUARSON, D, WITMANN, B. *et al.* (1986) Fetal echocardiography: results in high-risk patients. *Obstet Gynecol* **67**, 358–364.
21. CUCKLE, H. S., WALD, N. J., BARKAI, G. *et al.* (1988) First-trimester biochemical screening for Down's syndrome. *Lancet* **ii**, 851–852.
22. DUPONT, A., VAETH, M. and VIDEBECH, P. (1986) Mortality and Life Expectancy of Down's Syndrome in Denmark. *J. Ment. Defic. Res.* **30**, 111–120.

## Figures and Tables

**Figure 1 f1:**